# The impact of interrupting enzyme replacement therapy in late-onset Pompe disease

**DOI:** 10.1007/s00415-021-10475-z

**Published:** 2021-02-24

**Authors:** Stephan Wenninger, Kristina Gutschmidt, Corinna Wirner, Krisztina Einvag, Federica Montagnese, Benedikt Schoser

**Affiliations:** Department of Neurology, Friedrich-Baur-Institute, Ludwig-Maximilians University Munich, Ziemssenstr. 1, 80336 Munich, Germany

**Keywords:** Interruption of enzyme replacement therapy, Clinical outcome, Glycogen storage disease type 2, Pompe disease

## Abstract

**Background:**

Late-onset Pompe disease (LOPD) is a rare autosomal recessive disorder caused by mutations in the GAA gene, leading to progressive weakness of locomotor and respiratory muscles. Enzyme replacement therapy (ERT), administered every second week, has been proven to slow down disease progression and stabilize pulmonary function. Due to the COVID-19 pandemic in Germany, ERT was interrupted at our centre for 29 days. As reports on ERT discontinuation in LOPD are rare, our study aimed to analyse the impact of ERT interruption on the change in clinical outcome.

**Methods:**

We performed a prospective cohort study in 12 LOPD patients. Clinical assessments were performed after ERT interruption and after the next three consecutive infusions. We assessed motor function by muscle strength testing, a 6-minute-walk-test, pulmonary function tests, and adverse events. For statistical analysis, an estimated baseline was calculated based on the individual yearly decline.

**Results:**

The mean time of ERT interruption was 49.42 days (SD ± 12.54). During ERT interruption, seven patients reported 14 adverse events and two of them were severe. Frequent symptoms were reduced muscle endurance/increased muscle fatigability and shortness of breath/worsening of breathing impairment. After ERT interruption, significant deterioration was found for MIP_%pred_ (*p* = 0.026) and MRC_%pred_, as well as a trend to clinical deterioration in FVC_%pred_ and the 6MWT_%pred_.

**Conclusion:**

Interruption of ERT was associated with a deterioration in the core clinical outcome measures. Therefore, an interruption of ERT should be kept as short as possible.

**Supplementary Information:**

The online version contains supplementary material available at 10.1007/s00415-021-10475-z.

## Introduction

Pompe disease (glycogen storage disease type II, acid maltase deficiency) is a rare autosomal recessive neuromuscular disease that results from mutations in the GAA gene, which encodes the enzyme acid alpha-glucosidase (GAA) [1]. Reduced or absent GAA activity results in lysosomal accumulation of glycogen predominantly in muscle cells, but also smooth muscle cells and motor neurons [2]. Pompe disease is characterized by slowly progressive axial and proximal muscle weakness, combined with ventilatory insufficiency with the need for mechanical ventilation at later disease stages. Enzyme replacement therapy (ERT) has been approved since 2006, demonstrating a slowing of disease progression and stabilization of pulmonary function in clinical trials [3–5]. For adult Pompe patients (LOPD), ERT is usually administered by infusion every 2 weeks with 20 mg/kg body weight [5, 6].

COVID-19 causes a severe acute respiratory syndrome [7], and the pandemic and so called ‘lockdowns’ have caused enormous health, economic, and social consequences in many countries [8–10]. In March 2020, the government of Bavaria, Germany, announced a partial lockdown for university hospitals from mid of March 2020 until mid of April 2020 (29 days), which also covered the suspension of treatment of non-emergency therapies for inpatients and outpatients encompassing regular ERT infusions for LOPD patients.

Reports on the clinical impact of discontinuing ERT in LOPD are rare. Based on natural history studies, a yearly decline in pulmonary function (FVC) in sitting (1.0%) and supine position (1.3%) and in muscle strength (MRC) of 1.3% is estimated [11]. One retrospective study analysed seven patients with an ERT interrupted period between 3.1 and 59.3 months. Most of the patients showed a clinically meaningful decline in respiratory function and all patients in the 6-minute walk test. After ERT restart, a stabilisation in pulmonary function and stabilisation or improvement in the 6MWT was noted [12, 13]. However, reports on shorter treatment interruptions are lacking. Therefore, we analysed the clinical outcome of LOPD patients after short-term treatment interruption and after the ERT resumption for the next three consecutive ERT infusions.

## Methods

### Study setting and inclusion criteria

We conducted a prospective single-centre observational cohort study in patients with late-onset Pompe disease who discontinued ERT due to the COVID-19 pandemic in Munich, Germany. We assessed the clinical outcome after interruption and after the resumption of three consecutive ERT infusions. Inclusion criteria were (1) a genetically confirmed diagnosis for LOPD, (2) regular biweekly ERT administrations in the past 12 months, (3) interruption of ERT for more than 2 infusions, and (4) data of ≥ 3 retrospective yearly assessments before the COVID-19 pandemic lockdown. The assessments were performed within the national POMPE Registry programme, approved by the Ethics Committee of the Landesaerztekammer Rheinland-Pfalz (no. 7/04929). All patients gave written informed consent for participation in this registry. Yearly routine assessments within this registry programme and, if applicable, additional assessments for safety were performed within the standards of care and in accordance with ethical standards (Declaration of Helsinki 1975). During the lockdown period, all patients were contacted by telephone on the day of the planned ERT and asked for adverse events and disease-related symptoms. Prior to clinical assessments at *t*_0_ and *t*_1_ as well as prior to ERT infusions, patients were examined regarding temperature and vital signs and had to complete questionnaires regarding symptoms suggestive of any infection during the past ten days.

### Data collection

**Fig. 1:**
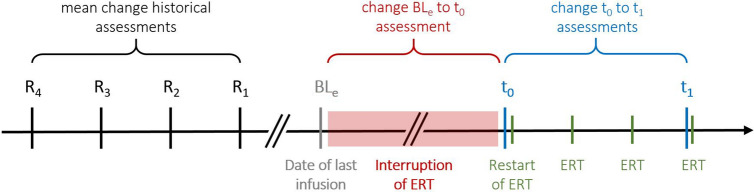
Timeline and schedule of assessments (BL_e_, *t*_0_ and *t*_1_)

For the evaluation of the clinical impact of ERT interruption, we calculated differences between the estimated baseline before ERT interruption (BL_e_), before (*t*_0_) and three infusions after the resumption of ERT (*t*_1_) (Fig. 1). We collected data from the following assessments: muscle strength test (Medical Research Council, MRC) of predicted %, the six-minute-walk-test in meters and predicted %, manometry (maximum inspiratory and expiratory pressure, both in cm H_2_O and predicted %) and spirometry (forced vital capacity in sitting and supine position) in litres and predicted % as well as FVC_drop_ (decrease from FVC_sit_ to FVC_sup_) for the assessment of diaphragmatic weakness.

### Estimated baseline (BL_e_)

Due to the unpredicted COVID-19-pandemic lockdown, sudden ERT cessation, different lengths of time between clinical assessments in standard of care and the beginning of the ERT interruption, we calculated an estimated baseline (BL_e_). This was based on historical assessments of the individual yearly progression of the disease and the days since the last assessment. This simple corrective mathematical approach made it possible to create a uniform and adjusted individual baseline for comparison (MC*y* = mean change of assessed value in %predicted):

$$ {\text{Mean yearly change}}\;MCy\;~\left[ {\% pred} \right] = \left( {\frac{{{\text{R}}3 - {\text{R}}4}}{{\Delta days{\text{~R}}4 - {\text{R}}3}}365 + \frac{{{\text{R}}2 - {\text{R}}3}}{{\Delta days{\text{~R}}3 - {\text{R}}2}}365 + ~\frac{{{\text{R}}1 - {\text{R}}2}}{{\Delta days{\text{~R}}2 - {\text{R}}1}}365} \right)/3 $$

$$ {\text{Estimated Baseline}}\;BLe\;\left[ {{\text{\% }}pred} \right] = {\text{R}}1{\text{~}}\left[ {{\text{\% }}pred} \right] + \frac{{{\text{MCy~}}\left[ {{\text{\% }}pred} \right]\left( {months{\text{~L}} - {\text{R}}1} \right)}}{{12}} $$

### Study procedures and clinical assessments

#### Muscle strength

The modified Medical Research Council (MRC) grading scale (0–5) was used to determine the skeletal muscle strength [14]. For the MRC sum score (maximum score 80), the following muscle groups were included: neck flexor and extensor, right and left shoulder abductors, elbow flexor and extensors, hip flexors and extensors, knee flexor and extensors. Values are presented as % of predicted.

#### 6-minute-walk-test

Distance walked in the 6-minute-walk-test (6MWT) as a measurement of functional endurance was recorded in meters and converted to the percentage of the predicted of normal. The test was performed according to the ATS guidelines [15, 16].

#### Pulmonary function assessments

Lung function test included spirometry (forced vital capacity, FVC) in an upright/sitting (FVC_sit_) and supine position (FVC_sup_) and manometry (maximal inspiratory pressure, MIP; maximal expiratory pressure, MEP). Values of FVC_sit_, MIP and MEP are presented as % of predicted, adjusted for age, height and sex, as applicable, according to published regression formulas [17, 18]. For assessment of diaphragm weakness, we calculated the reduction in FVC from sitting to the supine position, FVC_drop_ [19].

### Statistical analysis

To measure the impact of an ERT interruption, we compared the values of muscle strength, 6MWT, spirometry and manometry at three different time points (BL_e_, *t*_0_ and *t*_1_). Descriptive and explorative analysis was performed for demographic data and characteristics. The normal distribution was tested by Shapiro–Wilk-test. For all metric, normally distributed values, a quantitative linear model with paired two-sided students’ *t*-test was performed. The significance level was set at *p* < 0.05. For metric values not normally distributed we used the Wilcoxon-rank-test. Linear regression models were used to assess whether independent variables had an impact on the changes after ERT interruption, measured by MRC_%pred_, FVC_%pred_, MIP_%pred_, MEP_%pred_ and 6MWT_%pred_. For statistical analysis, we used SPSS statistics version 25.

## Results

### Patient’s characteristics

Thirteen patients consented to participate. Twelve patients met the inclusion criteria and were analysed (female 58.3%). One patient had to be excluded because the required minimum of three historical examinations had not been performed. All included patients received ERT every second week without relevant side-effects at our outpatient clinic for mean of 7.56 years (SD ± 4.79). Descriptive analysis of the cohort is summarized in Table 1. Due to two serious adverse events in two patients after *t*_0_, two patients were not able to perform assessments at *t*_1_.

**Table 1:** Patient characteristics

Patient characteristics (*n* = 12)			
	Mean ± SD	Median	range
Sex	Female: *n* = 7 (58.3%)		
Age at BL_e_ [years]	51.07 ± 16.62	50.17	24.60–80.00
Age at diagnosis [years]	41.07 ± 17.75	43.82	7.56–65.91
Age at start of ERT [years]	43.47 ± 17.36	44.19	12.16–66.73
Years on ERT until discontinuation [years]	7.56 ± 4.79	7.25	0.42–13.26
Duration of ERT interruption [days]	49.42 ± 12.54	42.00	36–70

### Adverse events

Seven patients reported 14 adverse events (AEs), two of them were classified as severe. The most frequent symptoms were reduced muscle endurance/increased muscle fatigability in six patients (50%), and shortness of breath/worsening of breathing impairment in three patients (25%). AEs and their description are summarized in Table 2. Twelve AEs have been classified as possibly related to the interruption of ERT. One female reported four AEs. Interestingly, this patient was one of the clinically mildest affected patients (MRC 95% predicted, FVC 84% predicted). AE No. 13 (fracture due to fall) and No. 14 (fracture due to fall) occurred in two males. Due to the description by the patients and evaluation of the event, these were classified as not related to the ERT interruption, whereas AE No. 7 (fall) in another female patient was possibly related to ERT interruption, as she complained about the deterioration of her muscle strength due to ERT discontinuation 14 days after her last infusion. None of the patients reported any symptom suggestible for COVID-19-infection during the period of ERT cessation and *t*_1_.

**Table 2:** Summary of reported adverse events after interruption of ERT or after restart of ERT

No. of reported AEs	AE no	Description	Patient no	Grade	Time of occurrence	Relation to ERT interruption
1	SAE 13	Bone fracture due to fall	6	Severe	22 days after restart of ERT	Not related
1	SAE 14	Bone fracture due to fall	7	Severe	14 days after restart of infusion	Not related
6	AE 1, 2, 4, 8, 10, 12	reduced muscle endurance/increased muscle fatigability	1, 2, 3, 5, 6	Moderate	Mean 33 days after last ERT	Possibly related
3	AE 3, 9, 11	Shortness of breath/ worsening of breathing impairment	2, 4, 6	Moderate	Mean 34 days after last ERT	Possibly related
3	AE 7, 13, 14	Fall	3, 6, 7	Moderate	AE 7: 14 days after last infusion AE 13: 22 days after restart of infusion AE 14: 56 days after restart of ERT	AE 7: possibly related AE 13: not related AE 14: not related
1	AE 5	increased exercise-related muscle pain	2	Moderate	15 days after last infusion	Possibly related
1	AE 6	Burning sensation in extremities during prolonged exercise and sitting	2	Mild	27 days after last infusion	Possibly related

### Impact of ERT discontinuation and restart of ERT on clinical outcomes

**Fig. 2:**
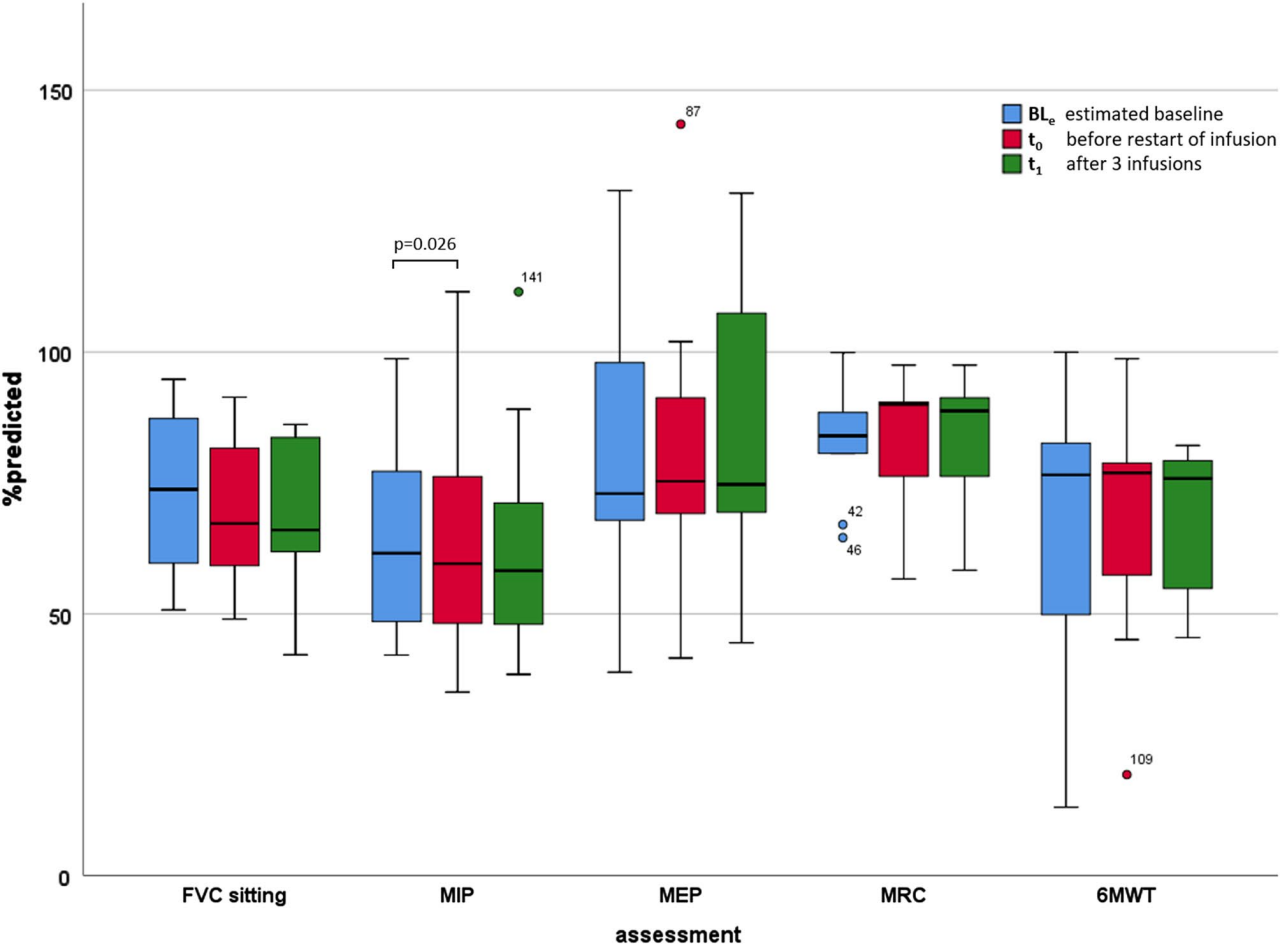
Assessments (FVC, MIP, MEP, MRC and 6MWT) in % predicted at BL_e_, *t*_0_ and *t*_1_. *BL*_*e*_ estimated Baseline, *t*_0_ before restart of ERT, *t*_1_ 3 infusions after restart of ERT, *FVC* forced vital capacity, *MIP* maximum inspiratory pressure, *MEP* maximum expiratory pressure, *MRC* medical research council, *6MWT* 6-minute walk-test. Coloured circles and numbers indicate outliers

**Fig. 3:**
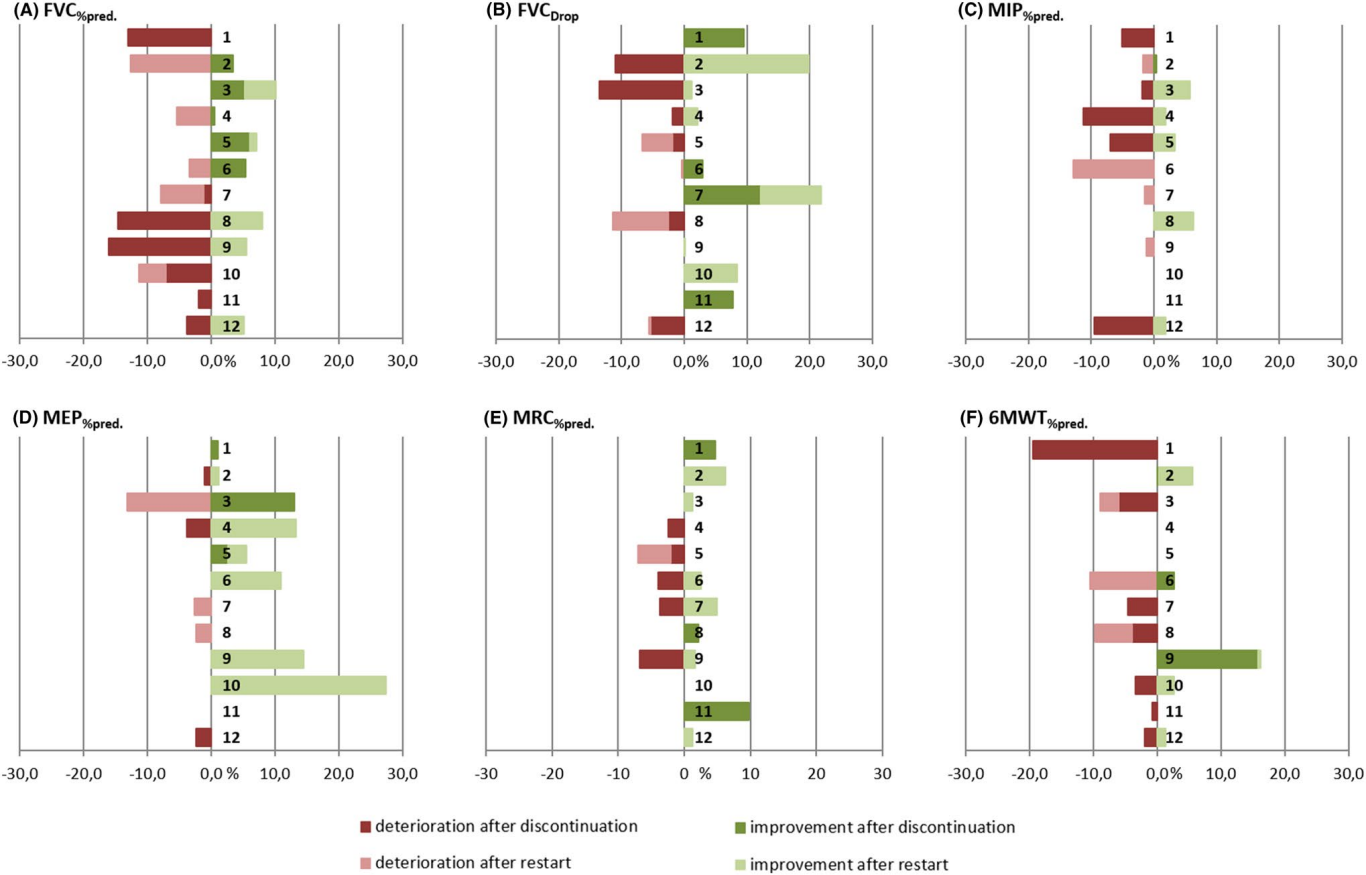
**a**–**f** individual changes in outcome measures. Changes are displayed in %change from BL_e_. The numbers per bar indicate the patient numbers. The red bars to the left indicate deterioration, green bars to the right indicate improvement. **a** FVC_%pred_ forced vital capacity %predicted; **b** FVC_Drop_ Drop of FVC sitting: supine position; **c** MIP_%pred_ Maximum inspiratory pressure % predicted; **d** MEP_%pred_ Maximum expiratory pressure % predicted; **e** MRC_%pred_ Medical research council % predicted; **f** 6MWT_%pred_ Six-minute walk-test % predicted

The mean time of ERT interruption was 49.42 days (SD ± 12.54; 36–70 days), and the mean time after restart of ERT between *t*_0_ and *t*_1_ was 43.90 ± 5.59 days (median 42.00; range 35–56). Except for MIP_%pred_, we could not detect a significant change in the assessments after ERT discontinuation (BL_e_–*t*_0_) or after the restart of ERT (*t*_0_–*t*_1_). In some of the patients, an insignificant improvement was observed in the following assessments: FVC%pred improved in four patients (3.4–6.1%), MIP%pred in one patient (0.4%), MEP%pred in two patients (2.7% and 13.1%), MRC%pred in three patients (2.1–9.9%) and in 6MWT%pred in three patients (0.3–15.8%) (supplements table S2). Outcome assessments at the three time points BL_e_, *t*_0_ and *t*_1_ are summarized in Table 3 and displayed in Figs. 2 and 3. Raw data per patient are summarized in supplementary tables S2 and S3. In linear regression modelling, we investigated the associations between changes in outcome measures from BL_e_ to *t*_0_ and the following independent parameters: the number of days of ERT interruption (Δ INT), the number of years on ERT (Δ ERT) and the age at the start of ERT (S ERT). The change of MRC_%pred_ from BL_e_ to *t*_0_ was associated with Δ INT (adjusted *R*^2^ = 0.91, *p* = 0.002) and in the overall model (adjusted *R*^2^ = 0.88, *p* = 0.021). Change in MIP_%pred_ was associated with all independent parameters in the overall model (ß_1_(Δ INT) + ß_2_(Δ ERT) + ß_3_(S ERT); adjusted *R*^2^ = 0.99, *p* = 0.002) (supplementary table S1). In other models, none was associated with the change of outcome measures from BL_e_ to *t*_0_.

**Table 3:** Mean values of spirometry, manometry, muscle strength test and 6MWT at BL_e_, *t*_0_ and *t*_1_

	BL_e_ ± SD (*n*)	*t*_0_ ± SD (*n*)	*p* value (BL_e_–*t*_0_)	*t*_1_ ± SD (*n*)	*p* value (*t*_0_–*t*_1_)
FVC_%pred_	72.92 ± 15.10 (12)	69.83 ± 14.05 (12)	0.207	69.68 ± 14.72 (10)	0.721
FVC_drop_ [%]	− 33.62 ± 10.18 (10)	− 31.67 ± 12.77 (12)	0.898	− 29.69 ± 13.21 (10)	0.315
MIP_%pred_	64.39 ± 20.83 (6)	63.60 ± 21.79 (12)	0.026	63.53 ± 22.98 (10)	0.910
MEP_%pred_	80.43 ± 30.93 (6)	79.93 ± 26.63 (12)	0.556	84.02 ± 27.46 (10)	0.185
MRC_%pred_	82.77 ± 11.82 (9)	82.51 ± 12.64 (11)	0.889	82.71 ± 13.72 (10)	0.217
6MWT_%pred_	65.15 ± 27.08 (11)	68.19 ± 22.45 (10)	0.453	67.37 ± 15.20 (7)	0.525

Values are provided as Mean ± SD (*N*)

*BL*_*e*_ estimated baseline, *t*_0_ before re-start of ERT, *t*_1_ 3 infusions after re-start of ERT; all variables at BL_e_, *t*_0_ and *t*_1_ were normally distributed, we used the students’ *t*-test for the comparison of paired samples in all cases, *%pred* percent of predicted of normal, *FVC* forced vital capacity, *MIP* maximum inspiratory pressure, *MEP* maximum expiratory pressure, *MRC* medical research council, *6MWT* 6-minute walk-test

## Discussion

This analysis was performed to evaluate the effects of a short-term interruption of enzyme replacement therapy in patients with genetically confirmed late-onset Pompe disease. Interruption of ERT in Pompe disease for a shorter duration has rarely been investigated. Today, however, emerging events such as the COVID-19 pandemic are becoming increasingly important for patients with chronic diseases who need to receive regular therapies. An interruption of these therapies may cause a clinical deterioration and, in a worst-case scenario, irreversible disease progression. Therefore, we investigated the clinical outcome after a short-term ERT interruption in LOPD. In particular, changes were calculated by a defined baseline examination followed by outcome measures. The unpredictable COVID-19 pandemic resulted in a partial lockdown in Munich, Germany. Thus an actual group or individual baseline was not available. Therefore, we calculated an estimated baseline (BL_e_) to create a uniform and adjusted baseline for all 12 patients comparing changes after this short-term ERT discontinuation. Using an estimated baseline including results of assessments over at least three previous years, we determined disease progression by avoiding interfering factors that could influence baseline values e.g. motivation, and concomitant diseases. Consequently, we assume that our calculated estimated baselines reflect useful and suitable values for our investigation.

In our cohort, the mean period of treatment interruption was 49.42 days (SD ± 12.54 days). Due to infusion schedules and patient´s concern of an increased risk of SARS-CoV2-infection in hospitals after the lockdown, interruption of ERT was up to 70 days in two patients. Overall, we saw a trend to deterioration after ERT interruption in objective assessments, predominantly in FVC_%pred_, FVC_Drop_, MIP_%pred_, and 6MWT_%pred._ Significant changes were only found for MIP_%pred_ and MRC_%pred_ in the regression models. Muscle strength, assessed by MRC_%pred_, showed significant deterioration in the linear regression model based on the number of days of ERT interruption (supplementary table S1). Both significant changes correspond to the most frequently reported adverse events by the patients.

From the patient´s perspective, we have noticed an increased rate of AE´s reported by the patients during therapy interruption, with the most frequent symptoms “reduced muscle endurance/increased muscle fatigability” and “shortness of breath/worsening of breathing impairment” in 75% of our patients. On average, the time from the last day of infusion and occurrence of the AE “reduced muscle endurance/increased muscle fatigability” was 33 days and for the AE “shortness of breath/worsening of breathing impairment” was 34 days, respectively. Both findings may be interpreted as related to ERT interruption. Six patients (50%) reported a “reduced muscle endurance or increased muscle fatigability” during ERT interruption. In seven patients (58%), we noted a deterioration in the six-minute-walk-test (6MWT_%pred_), but for the whole cohort, these changes were not significant. In the regression analysis, changes in the 6MWT_%pred_ were not associated with age of onset, years of ERT, or days of ERT interruption.

A slight but significant change was found for MIP_%pred_ after ERT interruption at the group level. The significant change in MIP_%pred_ may be explained by the fact that manometry of respiratory muscles detects changes earlier than FVC assessments [20, 21]. A worsening of breathing impairment was stated in three patients, but subgroup analysis did not reveal a significant deterioration in FVC_%pred_, MIP_%pred_ or MEP_%pred_ in those. Besides this, a deterioration of > 10% in FVC_%pred_ was found in three patients (25%), but only one of these patients (no. 9) reported a worsening of breathing impairment. In regression models, we found an association between MIP_%pred_ at the group level, however not for the number of days of interruption, the number of years on ERT, nor years since ERT start. When analysing the individual results, it was also noticeable that some patients had improved in some assessments after the ERT interruption. Even if the individual improvements are not statistically significant, this is still an interesting result. Although a linear trend is rarely observed in a clinical course especially in neuromuscular diseases, many factors may contribute to intermittent deteriorations or improvements. Frequent factors in daily clinical routine are affective components that may lead to an improved motivation after therapy cessation or learning effects in assessments that are performed more often than in routine diagnostics.

We cannot exclude a psychological factor contributing to the subjective deterioration in 75% of our patients. In the context of the COVID-19 pandemic, an increasing number of depressive and anxiety disorders across a high number of countries has been described [22], which certainly has an impact on both, subjective and objective assessment scales. Nevertheless, the reported adverse events of reduced muscle endurance and reduced/worsened pulmonary function correspond to the findings in objective measurements, even if some of them did not reach statistical significance. This highlights whether statistically significant deterioration in assessments truly reflects a clinical meaningful deterioration.

Our observations underscore the clinical benefits of regularly administered ERT in late-onset Pompe disease, which are based on clinical outcome measures and subjective reports from the patients. Correlating our findings and former reports, where a relevant decline in FVC and 6MWT after ERT interruption of 3.1 and 59.3 months was described [13], we can conclude that even a short-term interruption of ERT shows a trend to a clinical decline and should be avoided. This is not only relevant for objective outcome assessments, but also in terms of quality of life in this chronic progressive disease. Our results may also help to advise patients who may have to interrupt their regular treatment for a shorter period, e.g. holidays, travel, or hospitalization where ERT is unavailable. Further studies are necessary to evaluate changes in specific, validated patient-reported outcome measures covering depressive and anxiety symptoms.

## Conclusion

Emerging events, such as the COVID-19 pandemic, are becoming increasingly important for patients with chronic diseases who are at risk of not receiving their necessary therapy. Interruption of ERT in LOPD should be avoided or kept as short as possible, as our cohort showed a significant decline in MIP_%pred_, MRC_%pred_ and a trend to clinical deterioration in FVC_%pred_ and the 6MWT_%pred_ in objective outcome measures and an increased rate of adverse events. Further studies are necessary to evaluate the impact of interrupting ERT on clinical outcomes in LOPD more in detail.

## Supplementary Information

Below is the link to the electronic supplementary material.

Supplementary file1 (DOCX 23 KB)

## Data Availability

The anonymised participant data presented here are available upon request from the correspondent author (stephan.wenninger@med.uni-muenchen.de).
