# Supplementary material for: The impact of interrupting enzyme replacement therapy in late-onset Pompe disease
Source: J Neurol. 2021 Feb 24;268(8):2943–50. doi: 10.1007/s00415-021-10475-z (PMC7903209; doi:10.1007/s00415-021-10475-z)
Supplement: Supplementary file 1 — Supplementary file1 (DOCX 23 KB) [file 415_2021_10475_MOESM1_ESM.docx]

# Supplements

## Supplementary table S1: Linear regression model

|  | **overall model** | | | |  | **Δ INT** | | |  | **Δ ERT** | | |  | **S ERT** | | |
| --- | --- | --- | --- | --- | --- | --- | --- | --- | --- | --- | --- | --- | --- | --- | --- | --- |
| **Dependent variable** | **n** | **mean change Bl_e_-t_0_ [%]** | **adjusted R^2^** | **p-value** |  | **adjusted R^2^** | **ß** | **p-value** |  | **adjusted R^2^** | **ß** | **p-value** |  | **adjusted R^2^** | **ß** | **p-value** |
| FVC%pred | 12 | -3,09 | -0,09 | 0,578 |  | 0,017 | -0,33 | 0,301 |  | -0,07 | -0,178 | 0,581 |  | 0,035 | -0,351 | 0,191 |
| MIP%pred | 6 | -5,73 | **0,99** | **0,002** |  | -0,234 | 0,11 | 0,831 |  | 0,38 | -0,707 | 0,116 |  | 0,258 | 0,638 | 0,614 |
| MEP %pred | 6 | 1,57 | -0,43 | 0,720 |  | 0,000 | 0,45 | 0,374 |  | -0,09 | 0,356 | 0,488 |  | -0,168 | 0,256 | 0,526 |
| MRC %pred | 8 | -0,28 | **0,88** | **0,021** |  | 0,792 | **0,91** | **0,002** |  | 0,02 | -0,402 | 0,323 |  | 0,054 | 0,435 | 0,543 |
| 6MWT %pred | 10 | -2,15 | 0,09 | 0,361 |  | 0,055 | -0,40 | 0,251 |  | 0,07 | 0,414 | 0,234 |  | 0,127 | -0,473 | 0,359 |

Δ ERT: years on ERT before discontinuation; Δ INT: days of ERT interruption; S ERT: Age at start of ERT

## Supplementary table S2: Change after discontinuation of ERT per patient (BL_e_ - t_0_ in %)

| **Pt.No.** | **FVC_%pred_** | **FVC_Drop_ [%]** | **MIP_%pred_** | **MEP_%pred_** | **MRC_%pred_** | **6MWT_%pred_** |
| --- | --- | --- | --- | --- | --- | --- |
| 1 | -3.77 | -5.33 | -9.52 | -2.41 | na^2^ | -1.98 |
| 2 | -2.01 | 7.84 | na^2^ | na^2^ | 9.86 | -0.85 |
| 3 | -7.09 | na^2^ | na^2^ | na^2^ | na^2^ | -3.47 |
| 4 | -16.02 | na^2^ | na^2^ | na^2^ | -6.79 | 15.80 |
| 5 | -14.63 | -2.59 | na^2^ | na^2^ | 2.12 | -3.97 |
| 6 | -1.15 | 12.20 | na^2^ | na^2^ | -3.75 | -4.63 |
| 7 | 5.37 | 2.90 | na^2^ | na^2^ | -3.92 | 2.71 |
| 8 | 6.08 | -1.82 | -6.99 | 2.67 | -1.99 | na^2^ |
| 9 | 0.50 | -1.81 | -11.33 | -3.87 | -2.42 | na^2^ |
| 10 | 5.26 | -13.54 | -1.87 | 13.10 | na^2^ | -6.03 |
| 11 | 3.38 | -10.90 | 0.43 | -1.06 | na^2^ | 0.30 |
| 12 | -13.06 | 9.51 | -5.11 | 1.03 | 4.66 | -19.42 |

2: assessment not performed. %pred: percent of predicted of normal. FVC: forced vital capacity; MIP: maximum inspiratory pressure; MEP: maximum expiratory pressure; MRC: medical research council; 6MWT: 6-minute walk-test.

## Supplementary table S3: Change after restart of ERT per patient (t_0_ - t_1_ in %)

| **Pt.No.** | **FVC_%pred_** | **FVC_Drop_ [%]** | **MIP_%pred_** | **MEP_%pred_** | **MRC_%pred_** | **6MWT_%pred_** |
| --- | --- | --- | --- | --- | --- | --- |
| 1 | 5.11 | -0.23 | 1.90 | 0.00 | 1.25 | 1.34 |
| 2 | na^1^ | na^1^ | na^1^ | na^1^ | na^1^ | na^1^ |
| 3 | -4.27 | 8.42 | 0.00 | 27.33 | na^2^ | 2.61 |
| 4 | 5.59 | 0.17 | -1.27 | 14.49 | 1.67 | 0.39 |
| 5 | 8.01 | -8.79 | 6.26 | -2.40 | 0.00 | -5.82 |
| 6 | -6.80 | 9.65 | -1.44 | -2.60 | 5.00 | na^2^ |
| 7 | -3.39 | -0.38 | -12.85 | 10.87 | 2.50 | -10.49 |
| 8 | 1.04 | -4.84 | 3.39 | 2.90 | -5.00 | 0.00 |
| 9 | -5.40 | 2.17 | 1.92 | 13.34 | 0.00 | na^2^ |
| 10 | 4.92 | 1.21 | 5.80 | -13.16 | 1.25 | -2.93 |
| 11 | -12.60 | 19.83 | -1.71 | 1.19 | 6.25 | 5.26 |
| 12 | na^1^ | na^1^ | na^1^ | na^1^ | na^1^ | na^1^ |

1 Not available due to SAE; 2: assessment not performed. %pred: percent of predicted of normal. FVC: forced vital capacity; MIP: maximum inspiratory pressure; MEP: maximum expiratory pressure; MRC: medical research council; 6MWT: 6-minute walk-test.
